# Milk Antiviral Proteins and Derived Peptides against Zoonoses

**DOI:** 10.3390/ijms25031842

**Published:** 2024-02-03

**Authors:** Isabel Santos, Mariana Silva, Madalena Grácio, Laurentina Pedroso, Ana Lima

**Affiliations:** 1 Faculty of Veterinary Medicine, Lusófona University, 376 Campo Grande, 1749-024 Lisbon, Portugal; mrcamoesas19@gmail.com (M.S.); laurentina.pedroso@ulusofona.pt (L.P.); 2 CECAV—Centro de Ciência Animal e Veterinária, Faculty of Veterinary Medicine, Lusófona University, 1749-024 Lisbon, Portugal; 3 Instituto Superior de Agronomia, Universidade de Lisboa, Tapada da Ajuda, 1349-017 Lisbon, Portugal; madalenagracio@outlook.com

**Keywords:** milk, peptides, zoonoses, antiviral, one health

## Abstract

Milk is renowned for its nutritional richness but also serves as a remarkable reservoir of bioactive compounds, particularly milk proteins and their derived peptides. Recent studies have showcased several robust antiviral activities of these proteins, evidencing promising potential within zoonotic viral diseases. While several publications focus on milk’s bioactivities, antiviral peptides remain largely neglected in reviews. This knowledge is critical for identifying novel research directions and analyzing potential nutraceuticals within the One Health context. Our review aims to gather the existing scientific information on milk-derived antiviral proteins and peptides against several zoonotic viral diseases, and their possible mechanisms. Overall, in-depth research has increasingly revealed them as a promising and novel strategy against viruses, principally for those constituting a plausible pandemic threat. The underlying mechanisms of the bioactivity of milk’s proteins include inhibiting viral entry and attachment to the host cells, blocking replication, or even viral inactivation via peptide–membrane interactions. Their marked versatility and effectiveness stand out compared to other antiviral peptides and can support future research and development in the post-COVID-19 era. Overall, our review helps to emphasize the importance of potentially effective milk-derived peptides, and their significance for veterinary and human medicines, along with the pharmaceutical, nutraceutical, and dairy industry.

## 1. Introduction

There is a growing acknowledgment today that food serves not only as an energy source but also as a significant factor influencing health and well-being. Simultaneously, the food industry has undergone a substantial transformation to meet the evolving needs of consumers, leading to the development of novel bioactive and functional foods. A food product that provides nutrients, energy, and positively affects health by enhancing physiological responses and/or reducing the risk of disease is termed functional food [1]. The abundance of bioactive compounds in milk makes it an exemplary illustration of a functional food. The lifelong consumption of milk is currently a subject of debate, even though research is disclosing its advantageous effects at every life stage [2]. In addition to its fat and high-quality protein content, milk contains essential nutrients such as vitamin B12, riboflavin, selenium, magnesium, and calcium—nutrients with crucial roles in human health. Milk proteins, comprising caseins and whey proteins, serve as precursors to numerous bioactive peptides with a wide range of bioactivities, including antimicrobial, antihypertensive, antiatherogenic, immunomodulatory, antidiabetic, and antiviral properties [3,4,5,6,7,8,9,10]. This can be of particular significance in the post-pandemic era, where zoonotic diseases and antibiotic resistance present major global concerns. Indeed, the 2020–2021 COVID-19 pandemic has prompted us to reconsider and reshape many current healthcare paradigms, emphasizing a One Health view that transcends anthropocentrism to connect humans, animals, and the environment. While the holistic perspective on health has existed for some time, practical implementation proves intricate, as observed in the significant increase in infectious disease outbreaks attributed to animal viruses. Coupled with the resurgence of severe bacterial infections resulting from the currently developed antibiotic resistance, these challenges contribute to a growing number of epidemic outbreaks and novel pandemic scenarios [11,12,13]. Despite the crucial priority of diagnosing and predicting outbreaks, the absence of effective antiviral vaccines and therapeutics remains a significant concern. While the potential of enhancing immunity through nutrient-balanced foods has been shown against viruses [14,15,16], a novel trend is exploring the antivirals in foods by reevaluating the ancient practices of using plant parts and their active constituents as conventional curative agents for chronic infections, including viral diseases [17,18,19]. In the current surge of research on functional foods and bioactive compounds, we see a growing body of evidence supporting the potential of foods as supplementary therapies [17,20]; however, there is still limited information about the potential of milk’s proteins as antivirals, particularly within a One Health perspective. The consumer’s negative perception of milk might contribute to underestimating the potential of milk proteins and peptides. Considering the presence of antibacterial and antiviral peptides in fermented milk and whey, this aspect is becoming a compelling topic, advocating that milk bioactive peptides may present an opportunity worth pursuing. Within this context, our review primarily focuses on available milk peptides with antiviral and immune-stimulating properties, biologically active against various zoonotic viral infections. This could prove advantageous and supportive for ongoing and forthcoming research, shedding light on their potential efficacy against diverse zoonotic viral infections.

## 2. Milk Main Proteins and Structure

Milk has long been acknowledged as a nutrient-rich food product, playing a vital role not only in neonates but also in children’s growth and adults’ nourishment [21,22,23]. Comprising approximately 3.5% proteins, with 80% being caseins and 20% whey proteins, milk serves as a source of essential nutrients and defenses [8]. The caseins are α-, β-, and k-caseins, and the whey proteins comprise α-lactalbumin, immunoglobulins, and serum albumin, while a minor fraction is represented by lactoferrin, glycomacropeptide, lactoperoxidase, and lysozyme [24]. Tenascin C is a large, multimeric extracellular matrix glycoprotein that is found in a variety of tissues; it was also found in milk and presented marked bioactivity (see below) [25]. Finally, milk also contains a third class of proteins named mucins, which are present in the fat globule membrane [26]. Table 1 lists the main proteins in milk and their physicochemical characteristics.

The overall chemical composition of milk proteins consists of intricate structures that dictate their functions and properties. Understanding the detailed chemical makeup of milk proteins is fundamental for unraveling their diverse roles in nutrition and health. 

α-Lactalbumin, a water-soluble protein, is involved in lactose synthesis and possesses a compact fold with a metal-binding site. Rich in branched-chain amino acids, cysteine, lysine, and tryptophan, it plays a crucial role in supporting the growth and development of infants and children, with diverse bioactivities including immunological support, sleep regulation, mood enhancement, mineral absorption, and gastrointestinal function [10,29]. β-Lactoglobulin, present in milk from ruminant animals but nearly absent in human milk, is a lipocalin with a hydrophobic pocket capable of binding small hydrophobic molecules. It is a small protein, with excellent gelling and foaming properties. However, its potential allergenicity raises concerns for its use in food applications [30,31,32]. Lactoperoxidase, a heat-stable enzyme found in whey, comprises almost 1% of this fraction. Its high heat stability makes it an indicator of pasteurization efficiency in milk [33]. This enzyme catalyzes the oxidation of thiocyanate, producing an intermediate product with antimicrobial activity [34,35]. Caseins, predominant proteins in bovine milk, constitute most of its protein content. α-, β-, and k-caseins provide a complete amino acid profile, lacking cysteine but with a high amount of proline [27]. As phosphoproteins, caseins bind strongly to calcium, forming a micellar structure in milk formulas, crucial for water solubility and utilized in the cheese-making industry when coagulation occurs at lower pH [10,36]. 

These milk proteins not only possess a balanced amino acid composition and beneficial bioactivities but also act as precursors to various peptides with exceptional bioactivities released during protein digestion. This multifaceted contribution establishes milk as an invaluable asset for human health and nutrition.

## 3. Exploring Milk’s Peptides and Their Bioactivities

### 3.1. Peptide Production

**Figure 1:**
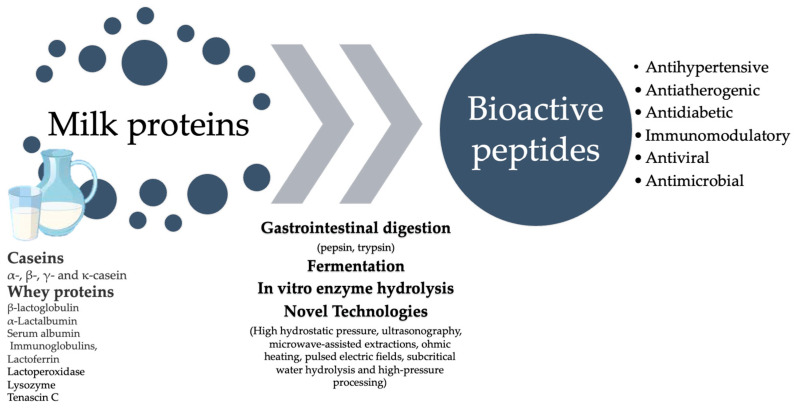
Overview of the possible mechanisms for releasing milk-derived peptides and related bioactivities.

Protein denaturation reveals buried peptide sequences and discloses the locations where proteolytic activity occurs. Bioactive peptides from milk proteins, such as casein or whey proteins, become active after hydrolyzation. These peptides, typically short amino acid sequences, are released during the enzymatic digestion of milk proteins or through fermentation processes. Milk-derived peptides, typically composed of 2–20 amino acids, can be generated either in vivo or in vitro. In vivo, peptides form through milk protein digestion via digestive enzymes like pepsin, trypsin, and chymotrypsin, responsible for protein hydrolyzation [37]. Alternatively, the in vitro simulation of gastrointestinal digestion involves using proteolytic enzymes like alcalase and thermolysin, combined with pepsin and trypsin. Milk-derived bioactive peptides can also be released during milk fermentation, employing various proteolytic microorganisms or their enzymes, such as *Lactococcus lactis* and *Lactobacillus helveticus* [8,37]. The biological activities of milk-derived peptides released in vivo or in vitro have been extensively studied and are illustrated in Figure 1.

Additionally, apart from conventional techniques such as enzymatic hydrolysis, fermentation, and in vitro digestion, novel technologies have emerged, aimed at enhancing the efficiency of bioactive peptide production. To effectively proteolyze parent proteins while maintaining functionality and bioactivity, many techniques such as high hydrostatic pressure, ultrasonography, microwave-assisted extractions, ohmic heating, pulsed electric fields, and subcritical water hydrolysis are being investigated [38]. High-pressure processing is another innovative strategy which facilitates hydrolysis by proteolytic enzymes [39,40]. Currently, these cutting-edge technologies are combined with enzymatic hydrolysis or microbial fermentation to reduce production costs while also enhancing yield, bioactivity, and efficiency [41,42]. 

Currently, dairy research benefits greatly from the versatile instrument of peptidomics, which goes beyond the study of protein digestion. In the dairy industry, peptidomics is used to find peptides in a variety of products, including kefir [43], yogurts [44], and cheeses [45,46,47,48]. In the pharmaceutical and nutraceutical fields, the potential of bioactive peptides is becoming increasingly better known due to their therapeutic use and their compatibility with medications without causing negative reactions [49]. These peptides are produced through the proteolytic cleavage of precisely sequenced amino acid chains. They are characterized by short peptide chains of two to twenty amino acids and a molar mass below 6000 Da [50,51]. This step of the food processing process is catalyzed by both endogenous and exogenous enzymes derived from plant, animal, and microbiological sources. Peptidome tools can be utilized in vitro to separate and monitor these targeted peptides throughout intricate proteolytic procedures [52]. 

### 3.2. Overview of Milk Protein and Peptide Bioactivities

Bioactive peptides derived from milk proteins have been gaining considerable attention for their potential health-promoting activities. Among the various bioactive peptides found in milk, some of the most studied include lactoferrin-, lactalbumin-, and casein-derived peptides. These peptides have been associated with a range of physiological activities, such as antimicrobial, antioxidant, immunomodulatory, and opioid-like properties [3,4,5,6,7,8,9,10,53]. For example, isoleucine-proline-proline (IPP) and valine-proline-proline (VPP) derived from β-casein and κ-casein, have demonstrated antihypertensive activities. Other milk-derived antihypertensive peptides, including those from αs1-casein, have shown blood pressure reduction in both in vitro assays and human clinical trials in Japan and Europe [7,54,55,56,57,58,59,60,61,62,63]. Other milk-derived peptides, particularly from casein and whey proteins, have shown promise in reducing cellular damage after the development of atherosclerotic plaques. These peptides exhibit antioxidant properties, and those derived from κ-casein and lactoferrin inhibit platelet aggregation, potentially preventing plaque rupture. Additionally, milk-derived peptides have demonstrated the ability to reduce cholesterol solubility in bile salt mixed micelles, leading to impaired cholesterol absorption [7,64,65,66,67,68]. 

Bioactive peptides from casein and whey have also shown potential inhibitory activities against α-glucosidase, responsible for carbohydrate absorption. Milk-derived peptides from various proteins, including β-casein, κ-casein, β-Lactoglobulin, α-lactalbumin, and lactoferrin, have demonstrated dipeptidyl peptidase 4 (DPP-4)-inhibitory activity, affecting blood glucose regulation. Some peptides, like isoleucine-proline-isoleucine, found in bioinformatic research, show promise in inhibiting DPP-4, with casein being rich in DPP-4-inhibitory peptides [69,70]. 

Casein- and whey-derived immunomodulatory peptides were also shown to stimulate immune activities, including human macrophage phagocytic activity, antibody synthesis, lymphocytes, and cytokine regulation. These peptides may prevent cancer cell growth by enhancing the activity of immune-competent cells. Peptides like lactoferricin B directly bind to neutrophils, displaying opsonin-like activity. Bovine β-casein-derived peptides affect phagocytosis in humans in vitro [71,72,73,74].

Alongside several bioactivities in milk proteins, one of the best known features of milk peptides are their antimicrobial activities. Milk-derived antibacterial peptides are a plentiful group with a molecular weight below 10 kD and have been extensively reviewed elsewhere [8,75,76,77,78,79]. These can exhibit antimicrobial activity against various pathogens, including *Escherichia coli*, *Aeromonas hydrophila*, *Salmonella* Typhi, *Bacillus cereus, Staphylococcus aureus, Yersinia enterocolitica*, and more [79]. Peptides like caseicidin, casocidin-I, and lactoferrin-derived peptides demonstrate antimicrobial effects against different bacteria and fungi [49,77,80,81,82]. Lysozyme, a minor constituent of whey, is present in both colostrum and human milk. Known for its ability to lyse bacterial cell walls, lysozyme is active against Gram-positive and Gram-negative bacteria [83,84]. They play a crucial role in controlling microbial infections, and their possible applications have been studied in vitro [85,86,87,88,89,90,91]. 

## 4. Antiviral Bioactivity of Milk Proteins and Peptides

### 4.1. Historical Survey

The understanding of the antiviral activities in milk peptides began with the early pivotal observation by Klebanoff and Ray [92] of the virucidal effect of the lactoperoxidase system against poliovirus (PV) and vaccinia virus. Fieldsteel (1974) [93] later demonstrated the presence of unknown antiviral substances in human milk, proving effective against arbovirus and murine leukemia virus. Matthews et al. (1976) [94] further validated the efficacy of proteins in human and bovine milk against arbovirus, rhinovirus, and influenza viruses. 

Since then, our knowledge of the antiviral potential of milk proteins and peptides has grown significantly. Most studies focused on bovine and human milk, with lactoferrin being one of the most reported proteins, as it demonstrates notable antiviral activities in humans and animals. For instance, apo- and holo-lactoferrin were shown to bind with canine herpes virus and surface receptors on the Madin–Darby canine kidney cells, preventing viral infection [95]. Human and bovine lactoferrin also inhibit herpes simplex virus 1 from entering the host cell and viral cell-to-cell spread in a dose-dependent manner [96,97,98,99]. The extensive antiviral effects of lactoferrin (LF) have since been extensively examined, with comprehensive reviews in the literature [9,100,101,102,103]. The most important antiviral properties have been ascribed to native lactoferrin and peptide derivatives such as lactoferricin and lactoferrampin [104,105,106], which, in some cases, showed strongly augmented antiviral effects compared to the native protein. Although lactoferrin stands out as the protein extensively researched for its antiviral properties, it is noteworthy that numerous proteins from milk exhibit antiviral effects [9]. 

For instance, human casein showed antiviral activity towards human immunodeficiency virus [107], human hepatitis B virus [108], and human rotavirus [109]. The other whey proteins also stand out with important antiviral actions, including against influenza virus A (H1N1), human cytomegalovirus, human immunodeficiency virus (HIV1), hepatitis B and C virus [110,111,112], avian influenza A (H5N1) [113], human rotavirus, human papilloma virus, and enterovirus [25]. 

Lactoperoxidase was also shown to present a wide range of antiviral activities against human immunodeficiency virus, herpes simplex virus 1, respiratory syncytial virus [114,115], and influenza virus [116]. Furthermore, certain peptides originating from casein and whey proteins have also been shown to stimulate the immune system or suppress host immune inflammation [117,118,119].

Bovine glycomacropeptide (GMP), also called caseinomacropeptide, is a milk-derived bioactive peptide that is released from κ-casein via enzymatic digestion, either physiologically or in industry during the cheese-making process [120]. This milk-derived bioactive peptide prevented the human RV infection of Rhesus monkey kidney in MA104 cell lines [109] and showed the ability to link to sialic acid, a known target of several viruses [121].

Peptides produced by α-lactalbumin showed anti-cytomegaloviral activity in MRC-5 fibroblasts [122]. These peptides also had antiviral activity against HSV-1 [123]. All these findings underscore the potential of milk proteins and milk peptides as a natural antiviral agent, highlighting their significance in both innate immunity and possible therapeutic applications.

### 4.2. Milk-Derived Antiviral Peptides Targeting Zoonotic Viruses

Animals often serve as reservoirs for viral zoonoses, diseases transmissible from animals to humans. While zoonotic viral diseases have been present in human populations since the inception of agricultural practices, they have gained increasing prominence as a global public health concern, particularly with recent epidemics like SARS-CoV-2 (COVID-19). Some of these diseases are categorized as “emerging infectious diseases” due to their newfound recognition or significant changes in their range and epidemiology. Notable zoonotic diseases include influenza, Ebola virus, West Nile virus, emerging coronaviruses, monkeypox, rabies, Zika, and Lyme disease [124]. Six out of every ten infectious diseases in humans are zoonotic, with many being viral. Therefore, it is imperative to enhance our capabilities to prevent and respond to these diseases, adopting a One Health approach. Finding new therapies and ways to prevent viral zoonoses is just as important as increasing efforts on surveillance and early detection, and with the rising amount of research demonstrating the potential of bioactive proteins and peptides produced from milk as antivirals, a vital opportunity arises to assess their usage in viral diseases with a focus on One Health. Indeed, several peptides derived from milk protein parents have shown potential for zoonotic viral diseases. Peptides from β-lactoglobulin and lactoferrin have gained attention for their potential effect against SARS-CoV-2, supported by numerous studies published in the past year [104,125,126,127,128]. Lactoferrin peptides have also been considered as immune boosters, which also aids in preventing viral infections [129,130]. In Table 2, we summarize the main zoonotic viral diseases to which antiviral activities were found in milk-derived proteins and peptides. Subsequently, we describe the antiviral effects of milk proteins and peptides against each zoonotic virus specifically.

**Figure 2:**
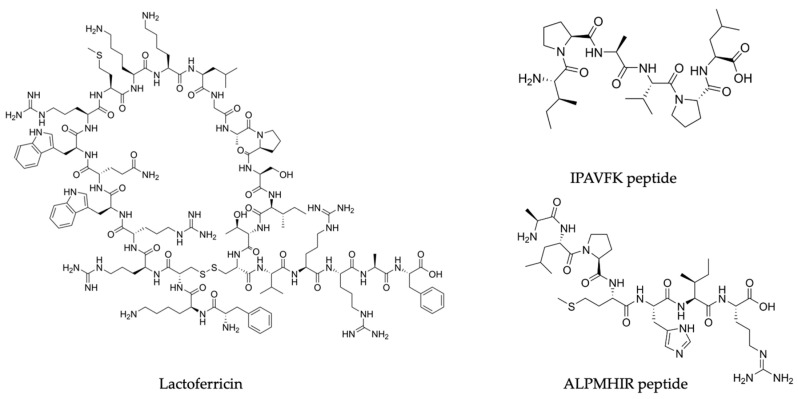
Molecular structure of three prominent milk-derived peptides with antiviral potential against zoonoses: lactoferricin and the ALPMHIR and IPAVFK peptides, derived from lactoferrin and β-lactoglobulin, respectively. Source: PubChem.

An overview of Table 2 shows us that the main participants in the antiviral activities against zoonoses were diverse and mainly consisted of whey proteins, mostly lactoferrin and β-lactoglobulin, with other key players such as α-lactalbumin, tenascin-C, lysozyme, glycomacropeptide, and lactoperoxidase. Information regarding the sequence of bioactive peptides is scarcer, although some well described peptides are lactoferrin-derived-peptides, particularly lactoferricin and the β-lactoglobulin-derived ALPMHIR and IPAVFK peptides. Interestingly, these peptides exhibit unique chemical structures essential to their bioactive functions: lactoferricin possesses a distinctive cationic amphipathic helical structure, whilst ALPMHIR and IPAVFK showcase specific amino acid sequences that contribute to their specificity [104,105,106,125,126,127,128]. Figure 2 depicts the molecular structure of some of these peptides. Subsequently, we describe the antiviral effects of milk proteins and peptides, against each zoonotic virus specifically.

#### 4.2.1. COVID-19

Severe acute respiratory syndrome (SARS) is a viral respiratory disease induced by SARS-associated coronavirus (SARS-CoV) which affected Asia, North America, and Europe in 2002–2003 [12]. In late 2019, a new type of coronavirus called SARS-CoV-2 caused a viral pandemic known as Coronavirus disease (COVID-19), which led to severe respiratory problems. Worldwide, researchers searched earnestly for molecules that could help create therapies for COVID-19 prevention and treatment. Coronaviruses enter target cells by fusing the membranes of the virus and the host cell, which is mediated by a viral spike glycoprotein (S protein) [131]. Earlier in 2020 a study showed that whey proteins from human, goat, and cow milk could inhibit SARS-CoV-2 entry and replication in Vero E6 and A549 cell lines, with an EC_50_ of about 0.13 mg/mL of total protein [132]. Among these molecules, milk and whey proteins, particularly lactoferrin, have been identified as one of the main antivirals present in milk and have been reviewed [133]. Lactoferrin was shown to present an array of activities against the virus, including a high affinity with the spike domain [134], competing for binding to an ACE2 receptor [135], and blocking the spike protein Furin-cleavage site [136]. It also hinders viral attachment by binding at the level of heparan sulfate proteoglycans [137,138] and binding to sialic acid [135]. Lactoferrin also reduces SARS-CoV-2 infectivity by inhibiting cathepsin L activity [139] and acts as an immune modulator of the antiviral immune response [140]. Lactoferrin was also shown to potentiate the effect of certain treatments towards SARS-CoV-2, such as remdesivir, hypothiocyanite anion, and the oral administration of liposomal Lf and oral zinc solution [138,141,142,143,144]. In addition to lactoferrin, other milk proteins were demonstrated to present antiviral activity against SARS-CoV-2 [145]; in particular, two β-lactoglobulin-derived peptides, produced by trypsin digestion, Ala-Leu-Pro-Met-His-Ile-Arg (ALMPHIR) and Ile-Pro-Ala-Val-Phe-Lys (IPAVFK), exhibited the ability to inactivate the virus via cathepsin inhibition and binding to the spike protein and also the host cell membrane receptor, showing great potential as a treatment for SARS-CoV-2 [129,139].

Interestingly, recent works have shown that milk-derived peptides are effective multi-targeted therapeutic candidates to treat SARS-CoV-2. Pradeep et al. [145] showed an interesting strategy involving the concurrent blockade of diverse pathways in the infectious cycle of SARS-CoV-2 to mitigate the COVID-19 threat. Through a combination of molecular docking, molecular simulation, heat mapping, and manual interpretation, the study developed a new strategy to identify a plethora of peptides capable of impeding the spread of the coronavirus [145].

#### 4.2.2. Human Immunodeficiency Virus (HIV)

Human immunodeficiency virus infection and acquired immunodeficiency syndrome (HIV/AIDS) are caused by a retrovirus known as the human immunodeficiency virus (HIV). HIV is a life-threatening infection that has spread globally since the first case was discovered, leading to a significant need for effective antiviral therapies. Milk-derived proteins such as human and bovine lactoferrin, modified β-lactoglobulin, α-lactalbumin, and lactoperoxidase have all demonstrated antiviral properties against human immunodeficiency viruses, mostly via the ability to link themselves to host cell receptors, inhibiting not only viral absorption but also the virus replication cycle [107,146,147,148,149,150].

Bovine lactoferrin and its derived peptide lactoferricin inhibit HIV-1 by acting on CXCR4 and CCR5 receptors [107,150]. Recently Berkhout et al. [107] showed that lactoferricin presents a lower inhibition when compared to lactoferrin, suggesting that other domains in the native protein may also aid in inhibition. Indeed, the apo form of bovine lactoferrin also inhibits HIV-1 replication in a different study [151]. Other whey proteins were also shown to inhibit the HIV virus. Modified β-lactoglobulin and α-lactalbumin were able to block the virus entry in the host cells due to interactions with the gp120 envelope protein [152,153], and Tenascin-C also displayed antiviral activity against HIV by interacting with its envelope domain, effectively neutralizing the retrovirus, and preventing transmission via breast milk [154]. In fact, the potency of Tenascin-C’s inhibition surpasses that of lactoferrin and is comparable to HIV-1-neutralizing monoclonal antibodies [155].

#### 4.2.3. Human Cytomegalovirus (HCMV)

Human cytomegalovirus (HCMV), also known as human herpesvirus 5 (HHV-5), is a virus that belongs to the *Herpesviridae* family. This virus can enter the human body through contact with mucous membranes or through blood components containing cells, as well as through stem cell/organ transplants [156]. In human cytomegalovirus infection, lactoferrin, lactoferricin, and methylated β-lactoglobulin and α-lactalbumin can inhibit virus replication and transcription by interacting with the viral genome [122,157]. Lactoferrin and lactoferricin display different mechanisms of action, being able to both interfere with the virus target cells and up-regulate the immune system, but also exerting a synergistic antiviral effect with cidofovir, an antiviral drug commonly used in patients with human cytomegalovirus [6,158,159,160,161,162]. Methylated β-lactoglobulin and α-lactalbumin, on the other hand, inhibit viral replication and transcription by interacting with the viral genome [122,133].

#### 4.2.4. Hepatitis B

Hepatitis B is an often-life-threatening liver infection caused by the hepatitis B virus (HBV). It is a major global health problem that can cause chronic infection and puts people at great risk of death from cirrhosis and liver cancer. The inhibition of hepatitis B (and C) viruses was shown by human and bovine lactoferrin (and peptide derivatives) as well as by α-lactalbumin, β-lactoglobulin, and lysozyme, via interaction between viral and cell proteins that interfere with virus entry and multiplication [123]. Here, lactoferrin, when saturated with zinc or iron, showed antiviral activity against this disease. As for the mechanism of action, lactoferrin, saturated with the positive ions mentioned above, was able to bind to several molecules of the host cell and, therefore, interfere with the virus’s ability to attach itself to the host cell and its ability to enter the host cell [108,163].

#### 4.2.5. Influenza

Influenza viruses seriously threaten global health, causing substantial morbidity and mortality, especially among individuals with weakened immune systems [164]. Whilst vaccination remains a cornerstone for infection control, its effectiveness is compromised by the rapid antigenic drift and the emergence of new viral subtypes [164]. Breastfeeding has been acknowledged for its protective role against respiratory and gastrointestinal infections in infants, so it would be expected that milk-derived peptides exert some activity against this virus [165]. Indeed, an early study found that lactoferrin exerts a protective effect against influenza-induced apoptosis by modulating caspase 3 function and impeding the export of viral ribonucleoproteins from the nucleus to the cytoplasm [166]. Subsequent research revealed a lactoferrin-derived peptide, bLf, which was shown to bind to the viral HA, inhibiting both hemagglutination and infection by influenza A viruses, of both group 1 and group 2 subtypes. Notably, bLf demonstrated binding to the HA2 subunit, which harbors the universally conserved HA epitope, explaining the broad-spectrum anti-influenza activity observed [164]. In a more recent study, Scala et al. [167] delved deeper into the inhibitory potential of lactoferrin-derived peptides against influenza virus infection and identified novel sequences derived from the C-lobe of bovine lactoferrin, demonstrating broad anti-influenza activity and the ability to prevent viral hemagglutination and infection at remarkably low concentrations [167]. Some in vitro studies have reported that lactoferrin and lactoperoxidase may also depend on other mechanisms, such as the inhibition of viral shedding, leading to the suppression of influenza virus A (H1N1) [116,168]. Bovine lactoferrin was found to interact with viral haemagglutinin, which resulted in the inhibition of virus-induced haemagglutination for influenza A virus. [164]; a similar mechanism of action was observed for glycomacropeptide in previous studies on influenza virus A [169].

#### 4.2.6. Zika and Usutu Virus

Zika virus is a mosquito-borne flavivirus that gained global attention due to its association with serious health concerns. Infection with Zika virus has been linked to neurological complications, including microcephaly in infants born to infected mothers, making it a significant public health issue [170]. A recent study [171] explored the antiviral properties of human milk at different stages of maturation against Zika virus and Usutu virus. The results indicated that human milk exhibited antiviral activity against both Zika virus and Usutu virus across all stages of lactation, and that extracellular vesicles and glycosaminoglycans played a role in the protective effect of milk, with no significant variations observed between colostrum, transitional, or mature milk. Mechanistic studies revealed that the mechanism was not due to the inactivation of the viral particles but was instead due to blocking the binding of both flaviviruses to cells.

#### 4.2.7. Rotavirus

Rotavirus is a global pathogen that is the major cause of severe diarrhea in infant mammals. In vitro tests showed the ability of human milk fractions to inhibit rotavirus replication, [172] particularly a mucin complex fraction containing the milk-fat globule membrane proteins MUC, lactadherin, and an unidentified 80-kDa whey protein [173,174]. While it was suggested that lactadherin might be responsible for the action of the mucin complex [173,174], MUC1 showed antiviral activity by inhibiting the replication of 3 human rotavirus strains [172]. Interestingly, only the human form of lactadherin could inhibit Wa rotavirus infection in vitro, apparently through a mechanism involving protein–virus interactions, which is dependent on the protein structure or the attached oligosaccharides [175]. According to Yolken et al. [173], the sialic acid present in lactadherin also plays a vital role in its antiviral action. Also, several whey proteins, including apo-lactoferrin (iron-free), homo-lactoferrin (carrying Fe3+), α-lactalbumin, and β-lactoglobulin, have demonstrated the capacity to hinder the attachment of rotavirus viral particles to host cellular receptors [176].

#### 4.2.8. Dengue Virus

Dengue is a significant mosquito-borne viral disease in tropical and subtropical regions. The responsible pathogen, dengue virus (DENV), consists of four distinctive serotypes: DENV-1, -2, -3, and -4. The infection can be mild or can result in clinically severe presentations, causing mild dengue fever, the more serious dengue hemorrhagic fever, or dengue shock syndrome [177]. A study in 2017 [178] reported the antiviral effect of bovine lactoferrin against DENV infection both in vivo and in vitro. Lactoferrin significantly inhibited the infection of the four serotypes of DENV and blocked binding between DENV-2 and the cellular membrane by interacting with heparan sulfate (HS), dendritic cell-specific intercellular adhesion molecule 3-grabbing non-integrin (DC-SIGN), and low-density lipoprotein receptors (LDLR) [178].

## 5. Overview of the Mechanisms Underlying Antiviral Activity

Proteins and particularly peptides are well known to play a vital role as a defense against bacterial or viral infections in all organisms [179]. However, unlike in antimicrobial activity, where we have an array of broad-range activities, only a few proteins and peptides have demonstrated antiviral properties, which are often very specific to the type of virus [12]. Overall, viruses are somewhere between living and non-living organisms; they are replicative, non-metabolizing, and lack self-generated energy [180,181]. If, on the one hand, an antiviral needs to avoid interfering with the host cell’s functions, on the other hand, RNA and DNA viruses have significant differences that make it challenging to develop broad-spectrum antivirals. As seen above, milk peptides and proteins have been shown to present diverse antiviral mechanisms that contribute to their potential in combating viral infections, and in some cases, such as lactoferrin and derived peptides, their activity can be broad-spectrum, albeit with different strategies altogether.

### 5.1. Main Targets

**Figure 3:**
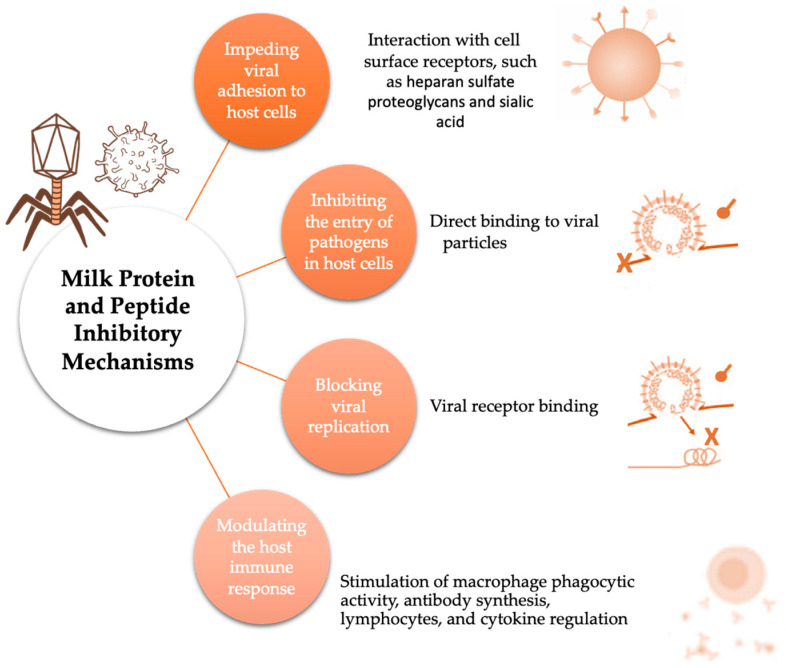
Mechanisms underlying the antiviral effects of milk proteins and peptides.

Overall, possible targets for antiviral therapies may include various steps in the lifecycle of a virus. These steps include attachment to host cells, fusion, and penetration, the uncoating and release of the viral genome, gene expression and multiplication, assembly and packaging, and the release of viral particles [182,183]. Although there is still little detailed information regarding most of the milk proteins and peptides’ mode of action, their main reported mechanisms involve either blocking the adhesion or the entry of pathogens into host cells, or, in fewer cases, inhibiting the viral replication itself or modulating the host immune response [168,184,185,186,187]. Figure 3 summarizes the main possible mechanisms behind the antiviral effects of milk proteins and peptides.

Regarding the first two, the most common mechanisms of milk proteins and peptides, much like other known antiviral peptides, are a) the inhibition of the fusion step to the host cell, interacting with the viral envelope and glycoproteins, and b) the blockage of viral entry by heparan sulfate interaction or binding to specific host cell receptors [12,168,184,185,186,187]. As for the third mechanism, some peptides can interfere with specific proteins or enzymes required for viral replication [12].

### 5.2. The Case of Lactoferrin

It is noteworthy to refer that, by far, lactoferrin has been the most extensively studied protein regarding antiviral mechanisms, as it has been shown to be a potent viral inhibitor in different studies and different viral species. As such, lactoferrin is possibly the most broad-range antiviral constituent of milk proteins and peptides. Two important paths are proposed to elucidate lactoferrin’s antiviral mechanisms:

(1) Interaction with cell surface receptors, such as glycosaminoglycans [188,189], which play a central role in virus docking onto target cells [190,191] and hence preventing them from binding to the cell host. Lactoferrin receptors have been identified in immune cells, including macrophages, lymphocytes, and dendritic cells [192,193].
(2) Direct binding to viral particles to prevent them from adhering to target cells [176,194,195,196].

**Figure 4:**
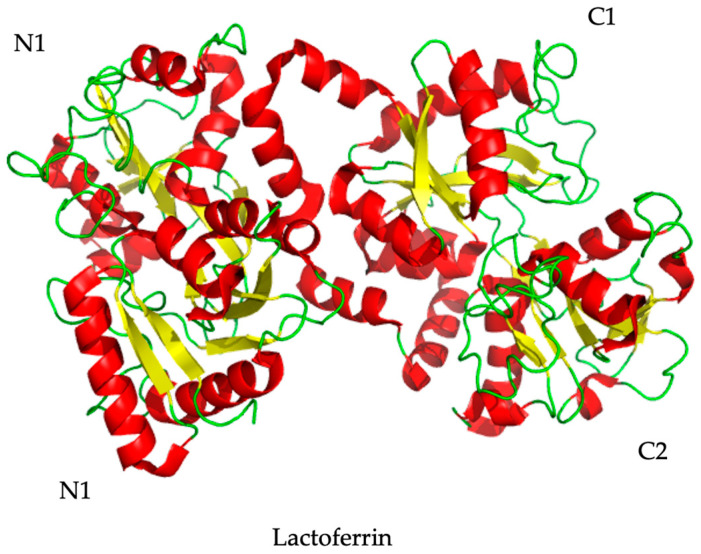
Representation of lactoferrin. The polypeptide chain forms two very similar and symmetrical globular leaves, the N lobe and the C lobe. The lobes can be further divided into two subdomains, represented in the figure by N1 and N2, and C1 and C2.

Overall, studies show that lactoferrin mainly acts by preventing host cell infection rather than inhibiting viral replication [102]. This is due mostly to the protein’s ability to bind iron, hence enabling both virus and host receptors [197], and to its amino acid sequence [147,148,198]. On the one hand, when compared to the apo-form, the iron-saturated lactoferrin has been suggested to have a higher affinity for eukaryotic cell receptors, due to a more compact shape [199]. Additionally, it exhibits increased resistance to proteolysis and denaturation [200]. On the other hand, lactoferrin has two symmetric globular lobes, as explained in Figure 4, each one including two domains, the N-lobe (N1 and N2) and the C-lobe (C1 and C2) [198]. Some studies confirmed that the different sequences in the N1 and 2 lobes of lactoferrin play an important part in the antiviral mechanism, as is the case with HSV-1 and -2 and cytomegalovirus [157,201]. Additionally, LF may induce indirect antiviral activity by upregulating the immune system. Orally administered lactoferrin was shown to upregulate macrophages and NK cells in both human volunteer trials and in vitro cell cultures [202], although the study lacks further in vivo confirmation.

It has also been suggested that lactoferrin interacts with numerous other components of human milk to mediate its range of antiviral and other effects [198]. This can in fact extend to all the remaining proteins and peptides. Indeed, the ability of the multi-target or “shotgun” model of several bioactive compounds as a higher efficient therapy against several diseases has become more popular recently [203]. By introducing milk bioactive peptides as “target-specific shotguns”, this idea is expanded upon with the goal of specifically inhibiting both host and viral targets. It seems therefore plausible to state that the numerous therapeutic effects of milk peptides, including antioxidant, anti-inflammatory, immunomodulatory, and analgesic activities might also be part of their overall antiviral mechanism.

## 6. Conclusions

Traditionally synonymous with health benefits, the perception of milk and dairy products has shifted over the years. While historical views once highlighted their health virtues, recent scientific studies may challenge certain assumed benefits. The high and regular consumption of milk and dairy products is now associated with alleged health problems. In response, the food industry is investing in developing processed vegetable drinks as alternatives to milk and dairy. However, amidst this trend, crucial aspects of milk and dairy that have dominated since humanity’s dawn are currently being overlooked. Indeed, recent findings suggest that milk peptides with antiviral properties hold great potential in aiding viral infections by (i) boosting the immune system, (ii) accelerating antiviral effectiveness against the infection, and (iii) decreasing other complications associated with the infection per se. The multifaceted nature of these antiviral mechanisms, combined with their many bioactivities, underscores the complex and promising role of milk peptides in the development of strategies to combat viral infections, providing possible avenues for therapeutic applications and the formulation of antiviral agents. However, although there is a growing body of evidence regarding lactoferrin and derived peptides, there is still much to know, particularly regarding whey proteins and caseins. There is also a lack of clinical tests to provide strong-based evidence of the real therapeutic effect of these proteins and peptides.

Although we can easily observe a growing interest in the antiviral activities of milk proteins and peptides in zoonotic diseases, particularly after COVID-19, future work should emphasize the mechanisms and feasibility of these antivirals in preventing or treating emerging diseases in both animals and humans, and in different clinical scenarios, so their potential becomes a reality. Possible strategies for their use as nutraceuticals or as functional ingredients in milk and dairy must also be pursued. As prevention stands alongside surveillance in the fight against emergent zoonotic diseases, the pursuit of milk bioactive peptides emerges as an opportune and valuable endeavor that should be further pursued.

## Figures and Tables

**Table 1:** Main proteins in milk and their physicochemical characteristics (adapted from [27,28]).

Protein	Molecular Weight (kDa)	Amino Acid Residues
αS1—casein	23.6	199
αS2—casein	25.2–25.4	207
β—casein	24	209
k—casein	19	169
β—lactoglobulin	18	162
α—lactalbumin	14	123
Imunoglobulins	25 (light molecular weight chain) + 50–70 (heavy molecular weight chain)	Variable
Albumin	66	582
Lactoferrin	80	690
Lactoperoxidase	70	612
Tenascin C	180–250	Variable
Lysozyme	14.3	130

**Table 2:** Antiviral effects of milk proteins and peptides against zoonotic viruses.

Virus	Protein/Peptide	Mode of Action
COVID-19	Lactoferrin and derived peptides	High affinity with the spike domain and blocking the spike protein competing for binding host cell Inhibition of viral attachment by binding to heparan sulfate proteoglycans and sialic acid Virus inactivation via cathepsin inhibition Adjuvant of pharmacological treatments
	β-lactoglobulin-derived peptides Ala-Leu-Pro-Met-His-Ile-Arg (ALMPHIR) and Ile-Pro-Ala-Val-Phe-Lys (IPAVFK)	Virus inactivation via cathepsin inhibition Binding to the spike protein
Human Immunodeficiency Virus (HIV)	Lactoferrin and derived peptides	Inhibition of HIV-1 adsorption to host cells by acting on CXCR4 and CCR5 receptors
	Tenascin-C	Interaction with viral envelope domains Prevention of transmission via breast milk
	Lactoperoxidase	Binding to host cell receptors, inhibiting viral absorption and the virus replication cycle More effective than lactoferrin
	Modified β-lactoglobulin and α-lactalbumin	Inhibition of viral entry into the host cells due to interactions with the gp120 envelope protein
Hepatitis B	Lactoferrin and derived peptides	When charged with iron or zinc, it inhibits viral adsorption to target cells
	α-lactalbumin, β-lactoglobulin, and lysozyme	Interaction with viral and cell host proteins blocking with virus entry and multiplication
Influenza	Lactoferrin	Modulating caspase 3 function
	Lactoferrin-derived peptides from the N lobe	Binds to host cells Inhibit both hemagglutination and infection
	Glycomacropeptide	Binds to host cells Inhibits both hemagglutination and infection
	Lactoperoxidase	Inhibition of viral shedding
Zika and Usutu Virus	Extracellular vesicles and glycosaminoglycans	Blocking the binding of both flaviviruses to cells
Rotavirus	Apo-lactoferrin, Homo-lactoferrin, α-lactalbumin, and β-lactoglobulin,	Binding to viral particles hindering the attachment to host cell receptors
	Mucin complex; human milk	Inhibits rotavirus replication
	Lactadherin	Binds viral receptors (important role of protein sequence and sialic acid)
		Human lactadherin is more efficient than the bovine form
	Unidentified 80-kDa whey protein	Unknown
Dengue	Bovine lactoferrin	Reduces infection Blocks binding to host cellular membrane by interacting with heparan sulfate, dendritic cell-specific intercellular adhesion molecule 3, and low-density lipoprotein receptors

## Data Availability

Not applicable.
